# Complete Genome Sequence of Strain JB001, a Member of *Saccharibacteria* Clade G6 (“*Candidatus* Nanogingivalaceae”)

**DOI:** 10.1128/MRA.00517-21

**Published:** 2021-06-10

**Authors:** Jonathon L. Baker

**Affiliations:** a Genomic Medicine Group, J. Craig Venter Institute, La Jolla, California, USA; University of Rochester School of Medicine and Dentistry

## Abstract

At least 6 highly diverse clades of *Saccharibacteria* inhabit the human oral cavity. However, all oral *Saccharibacteria* strains with currently available complete genome sequences or cultured isolates belong to clade G1, leaving clades G2 through G6 poorly understood. Here, a complete genome sequence of JB001, a clade G6 (“*Candidatus* Nanogingivalaceae”) *Saccharibacteria* strain, is reported.

## ANNOUNCEMENT

*Saccharibacteria* (formerly TM7) have reduced genomes, a small cell size, and appear to have a parasitic lifestyle, dependent on host bacteria (1–3). At least 6 major clades of *Saccharibacteria* (G1 through G6) inhabit the human oral cavity; however, all currently available complete genome sequences and cultured isolates belong to G1, leaving G2 through G6 quite poorly understood (4). Recent studies provided the first draft genome sequences from clades G3, G5, and G6 (5–8), which displayed major differences in encoded functional pathways, suggesting that the lifestyle and host dependency of the clades may be distinct (5, 6, 8). *Saccharibacteria* frequently lack what are considered “essential” core genes, which are typically relied upon to estimate the completion of draft genome sequences. Indeed, the sequence for the chromosome of TM7x, the first *Saccharibacteria* strain to be cultivated, had a completeness of 65%, according to CheckM (5). Therefore, obtaining complete genome sequences of *Saccharibacteria* is of special importance. In this study, Nanopore sequencing was used to deliver the first complete genome sequence of an oral *Saccharibacteria* strain outside clade G1, the G6 (proposed name, “*Candidatus* Nanogingivalaceae”) taxon JB001.

The draft assembly of JB001, Candidatus_Nanogingivalaceae_FGB1_strain_JCVI_27_bin.3, reported in 2021, was obtained from human saliva in Los Angeles, CA, USA, and fragmented into 67 contigs (6). Here, high-molecular-weight genomic DNA was extracted from the same saliva sample as used to obtain the original draft genome, using a phenol-chloroform-based protocol (9), and was examined for purity, size, and concentration using the TapeStation system (Agilent Technologies). The DNA was not sheared or size selected. A long-read library was prepared using a ligation sequencing kit (Oxford Nanopore Technologies) and sequenced on a GridION using an R9.4.1 flow cell (Oxford Nanopore Technologies). Base calling, quality control, and adapter trimming were performed using Guppy v4.0.11/MinKNOW v20.06.9 (Oxford Nanopore Technologies), resulting in 3,199,915 reads (*N*_50_, 13,719 bp). Two independent methods generated improved draft assemblies. (i) Human reads were removed using minimap2 v2.17-r941 (10), and the remaining long reads were assembled using meta-flye v2.8-b1674 (11). MegaBLAST v2.2.26 (12) was used to identify the circular JB001 contig within the metagenome assembly. (ii) Long reads mapping to the draft genome of JB001 were extracted using minimap2. These long reads, along with the short reads used to generate the original JB001 draft assembly, were used by Unicycler v0.4.8 (13) to obtain a draft genome of 4 contigs. Three short contigs were removed based on disparate GC content, coverage, and BLAST hits to other organisms (Anvi’o v7-dev [14]), leaving one circular contig. Trycycler v0.3.0 (https://github.com/rrwick/Trycycler) was used to develop a consensus assembly from the two draft assemblies. The resulting assembly was polished using Medaka v1.0.3 (https://github.com/nanoporetech/medaka), then Pilon v1.23 (15). Circulator v1.5.5 (16) was used to rotate the genome sequence start to *dnaA*. The scripts, parameters, and versions of the software tools used are available at https://github.com/jonbakerlab/JB001_genome_completion. Default parameters were used unless otherwise noted. JB001 was annotated using the NCBI Prokaryotic Genome Annotation Pipeline v5.1. The resulting chromosome was 662,051 bp with a GC content of 36.4% and is predicted to carry 687 genes. This resource will provide valuable information regarding the lifestyle and evolution of G6 *Saccharibacteria*.

### Data availability.

The complete genome sequence of JB001 has been deposited in GenBank under the accession number CP072208. The BioProject accession number for the genome is PRJNA624185. The raw Nanopore read library has been deposited in the Sequence Read Archive (SRA) database under the accession number SRX10387815. The short reads used to generate the original JB001 draft assembly are available in the SRA database under the accession number SRX4318838.
